# Use of the FallAkte Plus System as an IT Infrastructure for the North Rhine-Westphalian General Practice Research Network: Mixed Methods Usability Study

**DOI:** 10.2196/53206

**Published:** 2024-05-20

**Authors:** Arezoo Bozorgmehr, Simon-Konstantin Thiem, Dorothea Wild, Melanie Reinsdorff, Horst Christian Vollmar, Annika Kappernagel, Kathrin Schloessler, Sabine Weissbach, Michael Pentzek, Dorothea Dehnen, Julia Drexler, Beate Sigrid Mueller, Larisa Pilic, Lion Lehmann, Susanne Loescher, Elena Darinka Hohmann, Friederike Frank, Gülay Ates, Susanne Kersten, Achim Mortsiefer, Benjamin Aretz, Birgitta Weltermann

**Affiliations:** 1 Institute of General Practice and Family Medicine University Hospital Bonn University of Bonn Bonn Germany; 2 Institute of General Practice and Family Medicine Faculty of Medicine Ruhr University Bochum Bochum Germany; 3 Institute of General Practice/Family Medicine Medical Faculty University of Duisburg-Essen Essen Germany; 4 Institute of General Practice Faculty of Medicine University Hospital of Cologne Cologne Germany; 5 Institute of General Medicine Faculty of Medicine University of Münster Münster Germany; 6 Institute for Digitalization and General Medicine University Hospital Aachen Aachen Germany; 7 Institute of General Practice and Primary Care Faculty of Health Witten/Herdecke University Witten Germany

**Keywords:** primary care, general practice research network, physicians, feasibility study, IT infrastructure, usability, FallAkte, FallAkte Plus system, mixed methods usability study, North Rhine-Westphalian general practice research network, NRW-GPRN, Germany, German, universities, survey, questionnaire, participants

## Abstract

**Background:**

Primary care research networks can generate important information in the setting where most patients are seen and treated. However, this requires a suitable IT infrastructure (ITI), which the North Rhine-Westphalian general practice research network is looking to implement.

**Objective:**

This mixed methods research study aims to evaluate (study 1) requirements for an ITI and (study 2) the usability of an IT solution already available on the market, the FallAkte Plus (FA+) system for the North Rhine-Westphalian general practice research network, which comprises 8 primary care university institutes in Germany’s largest state.

**Methods:**

In study 1, a survey was conducted among researchers from the institutes to identify the requirements for a suitable ITI. The questionnaire consisted of standardized questions with open-ended responses. In study 2, a mixed method approach combining a think-aloud approach and a quantitative survey was used to evaluate the usability and acceptance of the FA+ system among 3 user groups: researchers, general practitioners, and practice assistants. Respondents were asked to assess the usability with the validated system usability scale and to test a short questionnaire on vaccination management through FA+.

**Results:**

In study 1, five of 8 institutes participated in the requirements survey. A total of 32 user requirements related primarily to study management were identified, including data entry, data storage, and user access management. In study 2, a total of 36 participants (24 researchers and 12 general practitioners or practice assistants) were surveyed in the mixed methods study of an already existing IT solution. The tutorial video and handouts explaining how to use the FA+ system were well received. Researchers, unlike practice personnel, were concerned about data security and data protection regarding the system’s emergency feature, which enables access to all patient data. The median overall system usability scale rating was 60 (IQR 33.0-85.0), whereby practice personnel (median 82, IQR 58.0-94.0) assigned higher ratings than researchers (median 44, IQR 14.0-61.5). Users appreciated the option to integrate data from practices and other health care facilities. However, they voted against the use of the FA+ system due to a lack of support for various study formats.

**Conclusions:**

Usability assessments vary markedly by professional group and role. In its current stage of development, the FA+ system does not fully meet the requirements for a suitable ITI. Improvements in the user interface, performance, interoperability, security, and advanced features are necessary to make it more effective and user-friendly. Collaborating with end users and incorporating their feedback are crucial for the successful development of any practice network research ITI.

## Introduction

Practice-based research networks (PBRNs) are collaborative enterprises between primary care practitioners and researchers [1] to address questions arising from daily practice in the setting where most health problems are diagnosed and treated. Historically, PBRNs originated in the late 1800s, and their tasks include systematic data gathering, observational studies, and engaging in research activities within primary care settings [2]. PBRNs have been successfully established in various countries worldwide and have proven effective in generating evidence-based knowledge, improving the quality of care, and fostering innovation in primary health care and its diverse populations [3-9]. However, for full functionality, PBRNs depend on a suitable IT infrastructure (ITI) [1], which is a key component for supporting tasks such as data management and collaboration within the network [10]. The ITI of a PBRN describes the software used to collect, integrate, store, and share data. The typical structure is a central platform (server) that integrates data from different practices and research institutions. Within the participating practices, electronic health records (EHRs) serve as digital repositories for patient health information, billing, and patient management [11]. In our study context, the relationship between EHR (within a practice) and ITI (for connecting practices) is of key interest. While the EHR is primarily concerned with care-oriented aspects of the health record, the ITI serves as a broader technological framework that supports research activities in the general practice network. This distinction is fundamental as we explore the impact of ITI usability on key outcomes and emphasize the importance of end-user involvement in ITI design.

In clinical settings, studies of EHR have demonstrated strong associations between perceived usability and important outcomes, including professional burnout [12] as well as performance [13]. A poor design of electronic records leads to ineffective data capture and workarounds, highlighting the importance of including end users in the selection and design of EHRs [14]. There are many studies evaluating the usability of clinical EHRs in the inpatient setting [15]. These show that providers in different countries hold very different views of the advantages and disadvantages of an EHR. In addition, workflow misalignments, poor usability, and irrelevant untimely information presentation are described [16-19]. Many of the problems described are also relevant for ITI-supported research networks, although the specific purposes and requirements for ITI differ from those for EHRs. In contrast, much less is known about ITIs, which aim at supporting general practice research networks. Available studies suggest that PBRNs require complex local customization and enhancements [20] and that users with different roles view their usability very differently [21]. A recent study of a Norwegian PBRN infrastructure identified several attractive features for a research interface but lacked a formal user evaluation [22].

The North Rhine-Westphalian general practice research network (NRW-GPRN) is a research project supported by the Federal Ministry of Education and Research to promote research in general practices [23,24]. To establish a suitable ITI for the network, this study aimed to answer two questions: (1) What are the general user requirements for an ITI suitable for supporting a general practice research network? (2) How do general practitioners (GPs), practice assistants (PrAs), and researchers rate the current usability of a commercially available IT solution system?

## Methods

### Study Design

The NRW-GPRN is 1 of 6 networks funded in Germany and is coordinated by the central unit Initiative of German Practice-Based Research Networks (DESAM ForNet) to facilitate collaboration at both national and international levels [25]. It consists of the 8 regional university institutes of general practice and family medicine located in Aachen, Bochum, Bonn, Dusseldorf, Essen, Cologne, Munster, and Witten/Herdecke, along with their respective research practice networks.

### Study 1: Questionnaire Survey Among Researchers to Identify Requirements for an ITI (Requirements Survey)

A questionnaire was developed by the authors on the basis of a previous research literature review [3-9] and derived six different dimensions for ITI:

1. Users: Who are the future users of the system?
2. Study types: Which study types should be supported?
3. Interfaces: What types of interfaces are needed for research purposes?
4. Data management: What requirements are necessary regarding data structures, data entry, and processing?
5. Access management: What requirements are necessary in terms of access, support, and monitoring?
6. Electronic case report form: What are the requirements for an electronic case report form?

The questionnaires, which consisted entirely of open-ended questions, were distributed via email to all participating institutes. Two researchers involved in the NRW-GPRN project independently categorized all responses using the 6 predefined dimensions. The dimensions were compared, and any discrepancies were addressed through discussion [26].

### Study 2: Usability of the FallAkte Plus System

#### Overview

**Figure 1:**
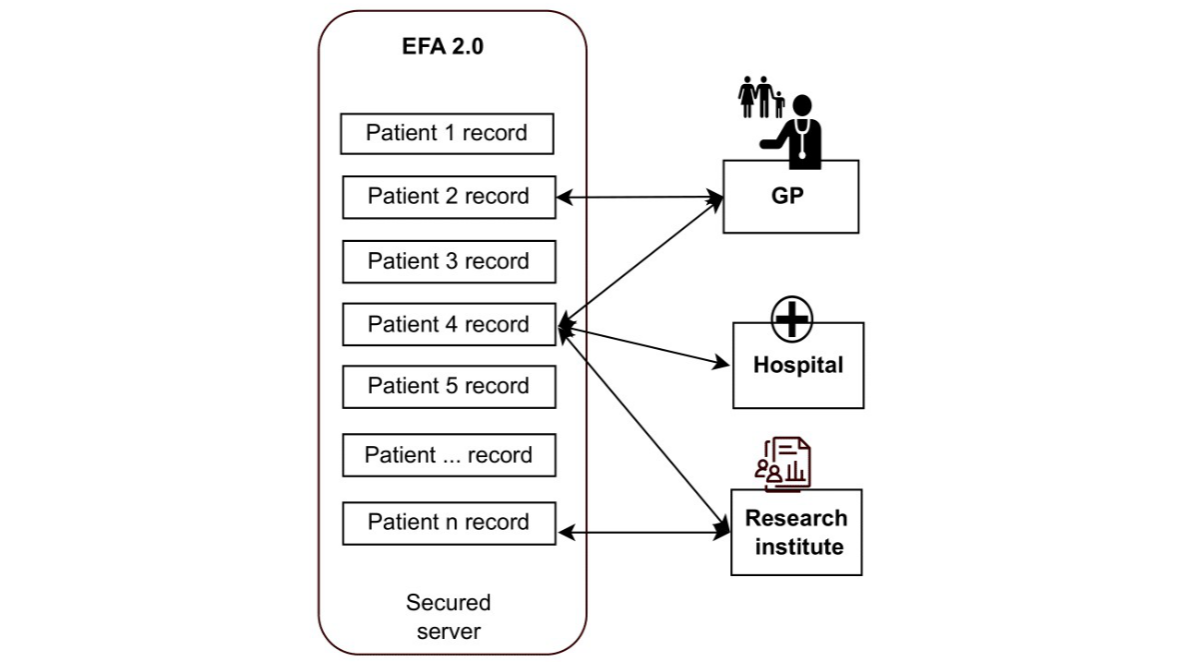
IT infrastructure and its connection to hospitals and practices, as well as relevant German research initiatives: the arrows illustrate data collection and data use by various stakeholders. EFA 2.0: Elektronische FallAkte 2.0; GP: general practitioner.

The FallAkte Plus (FA+) system emerged as a possible candidate due to its potential to comprehensively address the network’s technological requirements. The FA+ system is an implementation of the specification of the Elektronische FallAkte 2.0 (EFA 2.0), developed by the Fraunhofer Institute for Software and Systems Engineering. The EFA 2.0 specification is a blueprint for the implementation of medical data storage, taking international standards (Health Level Seven and Integrating the Healthcare Enterprise) as well as data privacy regulations into account (Figure 1).

It emphasizes decentralized storage of patient-related data within Germany and incorporates 2-factor authentication to enhance access security. The EFA 2.0 specification is publicly available and can be used under the condition of acknowledging the copyright [27]. FA+ is available on the market for service providers in the German health care system, including hospitals, practices, and physician networks [28,29].

Data protection in the FA+ system is ensured through various measures, including the use of a virtual private network, an association of statutory health insurance physicians (KV-Connect), and the telematics infrastructure. KV-Connect is the secure, privacy-compliant communication service provided by the Associations of Statutory Health Insurance Physicians (Kassenärztliche Bundesvereinigung) and the National Association of Statutory Health Insurance Physicians (Kassenärztliche Bundesvereinigung) in Germany [30]. Telematics infrastructure digitally connects all stakeholders in the statutory health insurance system, enabling secure cross-sectoral exchange of patient data among health care professionals [31]. The servers at the data center of Aachen University Hospital, where the FA+ system is hosted, hold technical inspection association level 3 certification, signifying rigorous testing for a high level of cybersecurity assurance and compliance with strict international standards. Additionally, they adhere to ISO (International Organization for Standardization) 27001-certified processes to ensure data safety. Compliance with the general data protection regulation in the European Union ensures that it meets the requirements for data security.

To assess the usability and acceptance of the FA+ system in its current stage of development, we combined qualitative and quantitative methods to gather data. The qualitative analysis aimed to achieve an in-depth understanding and captured nuanced feedback, while the quantitative analysis measured usability and acceptance.

#### Qualitative Evaluation: Think-Aloud Method

The think-aloud technique was used to explore the user experiences of the FA+ interface among researchers and practice personnel [32]. The researchers from 7 institutes and practice personnel from 10 practices were invited by mail and in person. Participants received emails with login credentials for registering in the FA+ system. They were further provided with a link to an on-demand tutorial video demonstrating the step-by-step process of installing and logging into the FA+ system. Digital sessions were subsequently arranged for groups of participants (n=1-3). Participants were instructed to verbalize each step they took while using the interface, including tasks such as installation, login, and use of the FA+ system. This test included a brief questionnaire on vaccines, serving as an example to assess the system’s usability and user experience. The interviews were transcribed and coded according to the grounded theory approach [33].

#### Quantitative Evaluation: Web-Based Questionnaire Survey

A web-based survey was developed using the German SoSci Survey platform (SoSci Survey Co). The survey was conducted among researchers from 7 institutes, and GPs and PrAs from 10 general practices. The survey consisted of three different instruments to assess the users’ experiences with the FA+ user interface: (1) the system usability scale (SUS) is a widely used and validated tool for assessing system usability [34]. Using a 5-point Likert scale, it consists of 10 questions and results in a score of 0 to 100, with higher scores indicating better perceived usability [34]. Systems with scores above 85 are considered “excellent,” those with scores 71-84 “good,” and those with SUS scores of 51-70 “Ok” [34]. (2) The German school-grade scoring system (1=excellent to 5=insufficient) was used to evaluate users’ experiences with the FA+ system as well as the training sessions using 17 different self-developed items. (3) The users’ technical affinity was measured with the 9-item affinity for technology interaction (ATI) scale [35]. This instrument was included to distinguish problems caused by the user interface from problems arising from users’ limited technical capabilities. A higher median technical affinity score suggests better ATI, while lower values suggest poorer ATI.

### Statistical Analysis

Descriptive statistics, such as absolute and relative frequencies, arithmetic means, and medians, were calculated for each variable using SPSS (version 27; IBM Corp). We abstained from collecting the sociodemographic characteristics of the participants because of the risk of potentially identifying data. As our study is nonclinical, trial registration was not required. To report this study, we used the CONSORT (Consolidated Standards of Reporting Trials) extension for feasibility studies and the COREQ (Consolidated Criteria for Reporting Qualitative Research) checklist [36].

### Ethical Considerations

Ethics approval for the usability evaluation of the existing FA+ system was obtained from the Ethics Committee of the Medical Faculty of the University of Bonn (reference 541/20). Informed consent was obtained from all participants and their legal guardians. For qualitative evaluation using the think-aloud method, participation in the session was considered as consent. For quantitative evaluation, data were collected anonymously, eliminating the need for informed consent. No compensation was paid to the participants. No reidentification is possible because data have been collected anonymously.

## Results

### Study 1: Questionnaire Survey Among Researchers to Identify Requirements for an ITI (Requirements Survey)

The questionnaire was answered by respondents from 5 of the 7 targeted research institutes. Details regarding the responses can be found in Textbox 1. Overall, the participants identified a wide range of requirements for an ITI.

**Results of the questionnaire survey among researchers to identify requirements for an IT infrastructure.**

**Users**

- Physicians
- Practice assistants
- Scientists or institutes
- Monitors
- Sponsors

**Study types**

- Quantitative studies
- Cross-sectional studies (eg, surveys)
- Prospective studies (cohort, observational, and clinical)
- Qualitative studies

**Interfaces**

- Exporting of data from various practice management systems
- Linking with electronic case report form (eCRF)
- Handling of different operating systems (Mac systems)

**Data entry, structures, and processing**

- Separation of study data and practice data
- Storage and reuse of study data
- Storage of consent forms
- Import of qualitative data
- Error management for data entry
- Import of paper surveys
- Pseudonymization or anonymization

**Access management**

- Access for new users or practices
- Training or troubleshooting format (digital vs on site)
- Access to new studies
- Availability (hours) of technical support
- Information for prospective users regarding new studies
- Training courses
- Monitoring for data safety

**eCRF**

- eCRF management
- Plausibility checks
- Regulatory compliance and audit capability
- Possibility of e-signature
- Support features
- Data management

**Other requirements**

- Coverage of costs after the funding phase
- Long-term use after completion of funded project

### Study 2: Usability of the Existing FA+ System

#### Qualitative Evaluation: Think-Aloud Method

A total of 36 respondents participated: 24 researchers from the NRW-GPRN-associated university institutes and 12 participants from general practices, including 8 family physicians and 4 PrAs. The think-aloud interviews had an average duration of 60 (range 30-75) minutes. The results of the think-aloud observation protocols are presented in Table 1.

All participants, including both researchers and practice personnel, found several aspects of the FA+ system to be highly favorable, including the overview of patients and studies, the ease and speed of data entry, and the search function. PrAs found the summary overview before creating a patient record particularly helpful, allowing for smoother patient management.

Despite the overall positive assessments, the participants identified some challenges and issues. Researchers highlighted specific concerns related to data protection (password requirements too lax and patient confidentiality). Additionally, the system erroneously marked emails as “read” after accessing the inbox, which could lead to communication errors. Participants, including researchers and practice personnel, raised critical concerns regarding data security and protection. They reported that patient data, including records not created by the user, were visible to unauthorized individuals. This included sensitive information such as the patient’s surname, first name, and date of birth. This raised significant concerns about the confidentiality and privacy of patient data within the system, necessitating urgent attention and improvements in data security measures to safeguard sensitive patient information.

Practice personnel from various practices experienced difficulties with the user interface when managing multiple studies within the FA+ system, indicating room for improvement in this aspect. However, several concerns were raised by participants within the scope of the user training. A common issue mentioned by all participants was the speed of the video explanation, which was considered too fast and at times confusing. Users requested a navigation function enabling them to quickly jump to the “relevant” sections of the video. Practice personnel also expressed concerns about the training materials, noting that they were not tailored to their specific roles. They found the instructions to be overly complex, hindering their ability to effectively use the training resources.

**Table 1:** Usability and acceptance of the FallAkte Plus system: think-aloud approach results by identified requirements.

Dimension	Benefits	Problems or concerns
User or eCRF^a^	Delegation to practice assistants	N/A^b^
Study types	User interface for multiple studies	N/A
Interfaces	N/A	Certificate was clicked instead of imported Copying password with spaces did not work Certificate was blocked by local IT Back button was missing Installation of security certificate for Mac systems
Data entry, structures, and processing	Overview of patients or studies positively assessed Easy or fast data entry in questionnaires	Changes after data entry in questionnaires User interface for questionnaire not ideal Data protection: patients’ data, including records not created by the user, are visible: surname, first name, and date of birth Records of patients not belonging to this study Data protection issues: records of patients not belonging to this study
Access management	Technical help available	N/A
Other requirements	Clearly structured and easily understandable processes Search function Summary overview before creating a record for a patient	Slow system speed Simple password was allowed Mails marked as “read” after checking inbox

^a^eCRF: electronic case report form.

^b^N/A: not applicable.

#### Quantitative Evaluation: Web-Based Questionnaire Survey

The questionnaire was voluntary; hence, a total of 21 of 36 participants completed the survey (response rate=60%). The median system usability score was 60/100 (IQR 33.0-85.0) points. There were notable differences in the SUS score among the groups: the GPs or PrAs rated FA+ as user-friendly with a median SUS score of 82 (IQR 58.0-94.0) points, indicating nearly good usability. With a median score of 44 (IQR 14.0-61.5) points, NRW-GPRN researchers gave the system a poor rating. The detailed results can be found in Table 2.

The results from users’ experiences with the FA+ system using the German school grading system showed a similar discrepancy between GPs or PrAs and researchers. Among all participants, the functionalities of the FA+ system (GPs or PrAs: median 1.5, IQR 1.0-2.8 and researchers: median 3.0, IQR 2.8-4.0) and the preceding training (GPs or PrAs: median 1.0, IQR 1.0-2.0 and researchers: median 2.5, IQR 1.0-3.0) received excellent to satisfactory ratings. The researchers rated the following items as good: creating a folder for new study participants (median 2.0, IQR 2.0-2.0), computer settings (median 2.0, IQR 2.0-3.0), and filling out the questionnaire (median 2.0, IQR 2.0-2.8). The researchers were more critical and rated 3 items (data protection, data security, and speed of the system) with scores of 4.5, 4.0, and 4.0, respectively. However, compared to GPs and PrAs, the researchers rated these aspects lower by 2.5, 2, and 2 grades, respectively (Table 3).

The users’ median technical affinity score was 3.0 (IQR 3.0-4.0) for the GPs or PrAs and 4.0 (IQR 3.0-4.0) for the researchers. In the total sample, the median score was 3.5 (IQR 3.0-4.0). Categorizing the results, approximately 4 (17%) of both GPs or PrAs and researchers showed a high technical affinity, while the majority of all participants (n=17, 71%) fell into the medium technical affinity group. For more details, refer to Table 4.

**Table 2:** SUS^a^ results for the FallAkte Plus system by researchers from institutes and practice personnel (SUS scale scores ranged from 1=strongly disagree to 5=strongly agree; range 0-100).

			GPs^b^ or PrAs^c^ (n=11)		Researchers (n=10)		Total (n=21)
**SUS score**							
	Mean (SD)	72.7 (25.5)		38.8 (23.3)		56.6 (29.5)	
	Median (IQR)	82.0 (58.0-94.0)		44.0 (14.0-61.5)		60.0 (33.0-85.0)	
	Range	16.0-96.0		6.0-70.0		6.0-96.0	
**SUS in categories,** **n (%)**							
	System has significant usability problems	3 (27)		7 (70)		10 (48)	
	System borderline to good	2 (18)		3 (30)		5 (24)	
	System good to excellent	6 (55)		0 (0)		6 (29)	
	System perfect, no usability problem	0 (0)		0 (0)		0 (0)	

^a^SUS: system usability scale.

^b^GP: general practitioner.

^c^PrA: practice assistant.

**Table 3:** Users’ experiences with the FA+^a^ system as well as the training sessions using the German school grading system (1=excellent to 5=insufficient).

		GPs^b^ or PrAs^c^ (n=12)		Researchers (n=14)		Total (n=26)	
		Median (IQR)	Missing, n (%)	Median (IQR)	Missing, n (%)	Median (IQR)	Missing, n (%)
**Functionalities of the FA+ system**							
	Overview of study participants	1.5 (1.0-2.8)	0 (0)	3.0 (2.8-4.0)	8 (57)	2.0 (1.0-3.0)	8 (31)
	Creating folders for new study participants	1.0 (1.0-2.8)	0 (0)	2.0 (2.0-2.0)	6 (43)	2.0 (1.0-2.0)	6 (23)
	Computer settings	1.0 (1.0-3.0)	1 (8)	2.0 (2.0-3.0)	9 (64)	2.0 (1.0-3.0)	10 (39)
	Completing the questionnaire	1.0 (1.0-2.0)	1 (8)	2.0 (2.0-2.8)	6 (43)	2.0 (1.0-2.0)	7 (27)
	Registration process	2.0 (1.0-2.8)	0 (0)	3.0 (2.0-4.0)	7 (50)	2.0 (1.0-3.0)	7 (27)
	Installation of the security certificate	2.0 (1.0-2.0)	0 (0)	3.0 (2.0-4.0)	4 (29)	2.0 (1.8-2.3)	4 (15)
	Search function	1.0 (1.0-3.0)	2 (17)	3.0 (2.0-4.0)	4 (29)	2.0 (1.0-3.0)	6 (23)
	Data protection	2.0 (1.0-3.0)	0 (0)	4.5 (4.0-5.0)	6 (43)	3.0 (2.0-4.8)	6 (23)
	Data security	2.0 (1.0-3.0)	0 (0)	4.0 (3.0-5.0)	7 (50)	3.0 (1.0-4.0)	7 (29)
	Speed of the system	2.0 (1.3-3.8)	0 (0)	4.0 (2.8-4.3)	4 (29)	3.0 (2.0-4.0)	4 (15)
	Time between registration and first login	1.0 (1.0-2.0)	1 (8)	3.0 (2.0-3.0)	9 (64)	1.5 (1.0-3.0)	10 (39)
**Overall rating of FA+**							
	How do you rate the FA+ concept?	2.0 (1.0-2.0)	0 (0)	3.0 (2.5-4.0)	4 (29)	2.0 (1.0-3.0)	4 (15)
	How do you rate the FA+ user interface?	2.0 (1.0-2.8)	0 (0)	3.0 (2.0-4.0)	4 (29)	2.0 (2.0-3.0)	4 (15)
	How do you rate the FA+ system overall?	2.0 (2.0-2.0)	0 (0)	3.0 (2.5-4.0)	5 (36)	2.0 (2.0-3.0)	5 (19)
**Training**							
	Clarity of responsibility of the physicians and PrAs	2.0 (1.0-3.5)	7 (58)	4.0 (3.5-4.5)	9 (64)	3.5 (1.8-4.0)	16 (62)
	Documentation, instructions, links, and help	1.0 (1.0-2.0)	0 (0)	3.0 (2.0-3.0)	6 (43)	2.0 (1.0-3.0)	6 (23)
	FA+ video tutorial	1.0 (1.0-2.0)	0 (0)	2.5 (1.0-3.0)	4 (29)	1.5 (1.0-3.0)	4 (15)

^a^FA+: FallAkte Plus.

^b^GP: general practitioner.

^c^PrA: practice assistant.

**Table 4:** Results of the users’ technical affinity score by researchers and practice personnel (1=completely disagree to 6=largely agree).

		GPs^a^ or PrAs^b^ (n=12)	Researchers (n=14)	Total (n=26)
**Technology affinity score**				
	Mean (SD)	3.5 (1.2)	3.7 (1.0)	3.6 (1.0)
	Median (IQR)	3.0 (3.0-4.0)	4.0 (3.0-4.0)	3.5 (3.0-4.0)
**Affinity for technology in categories, n (%)**				
	High (category 3)	2 (17)	2 (17)	4 (17)
	Medium (category 2)	8 (67)	9 (75)	17 (71)
	Low (category 1)	2 (17)	1 (8)	3 (13)

^a^GP: general practitioner.

^b^PrA: practice assistant.

## Discussion

### Principal Findings

This study identified several requirements for an ITI. The requirements for ITI we found are similar to but not identical to the functional components of PBRNs as identified in the study of Peterson et al [3], likely reflecting the different viewpoints of future users as in our study compared to IT administrators.

Due to privacy concerns, lack of integration, and insufficient support of various survey and study formats, it was determined that, in its current state, the commercially available FA+ system is unsuitable for the NRW-GPRN network. Perceived health record usability is a crucial component in the acceptance, use, and performance of physicians and other users [15]. The user experience of a record system needs to be closely aligned with user information retrieval and processing to be perceived as useful [14].

When comparing this study with the PBRN in Norway (PraksisNett) [22], we find similarities in the structural elements, notably resembling the FA+. However, it is crucial to highlight that while structural similarities exist, the focus and feasibility differ substantially. Our focus was on evaluating the fit of an existing system rather than a description of a primary research network.

We observed differences in SUS scores between GP personnel and researchers. GP personnel rated FA+ as user-friendly, achieving a median SUS score of 82 (IQR 58.0-94.0) points, indicating nearly good usability. In contrast, NRW-GPRN researchers gave the system a poor rating with a score of 44 (IQR 14.0-61.5) points. These variations in SUS scores could be attributed to existing differences in user requirements and technical proficiency between the 2 professions. Since no similar IT approaches have been studied in German general practices, a direct comparison of their usability with other IT solutions is not feasible. Moreover, the literature reports only a few studies on user-oriented usability evaluations of newly developed electronic tools supporting patient-centered care management [37-40].

The perception of the FA+ varied among GPs, PrAs, and researchers, potentially influenced by differences in technical proficiency between the subgroups. This divergence could be attributed to differences in technical proficiency between the 2 subgroups. Notably, the practice personnel, as participants, reported higher levels of technical proficiency compared to researchers, as indicated by self-reported measures. However, it is important to acknowledge that a self-reporting bias may have influenced these assessments [41]. This discrepancy in technical proficiency may explain why GPs or PrAs were more receptive to using ITI systems like FA+ compared to researchers. Nevertheless, it is crucial to note that technical affinity is just one aspect that can impact an individual’s use and evaluation of technology. Other factors, including system complexity, level of training and support provided, and users’ prior experience with similar systems, can also influence their perception of a system’s usability, as supported by previous research [42].

Finally, this study confirmed the value of obtaining end-user feedback to ensure that the ITI is compatible with users’ cognitive load and organizational aspects [14]. To address the existing challenges and enhance the FA+ system’s suitability as an ITI, the study findings were shared with the FA+ developer company, which has already started implementing improvements based on the insights gained from this study. These improvements include enhancing system speed, improving search functionality, enhancing data protection and security standards, and refining certificate management.

### Strengths and Limitations

In an effort to obtain comprehensive insights, this study adopted a holistic approach by involving GPs, PrAs, and researchers. It is innovative in evaluating a commercially available ITI in Germany, providing a robust qualitative and quantitative assessment. The mixed methods approach offers nuanced insights. Addressing both potential requirements and current usability, the study sheds light on the ITI’s strengths and weaknesses, facilitating targeted improvements. However, its focus on testing the FA+ system in North Rhine-Westphalia limits its generalizability to all of Germany. The small convenience sample may restrict broader applicability, necessitating caution when extrapolating findings to a larger population.

Future studies should evaluate the usability of various ITIs to facilitate a connection between primary care physicians and research units. Additionally, future studies should explore methods to enhance the usability and acceptance of the FA+ system. Intervention studies that specifically target improving usability would be particularly valuable in this regard. By implementing interventions and evaluating their impact, researchers can identify areas for improvement and enhance the overall user experience of ITI systems.

### Conclusions

This study indicates that the FA+ system does not fulfill all the requirements of GPs, PrAs, and researchers as a suitable ITI system. There is a significant demand for pilot information systems that can potentially be used in general practice research networks and undergo thorough testing by future users. Such a system should address the requirements of practices and researchers, as evaluated in this study, while also seamlessly integrating into clinical practice workflows. We hope that our findings can contribute to building such systems.

## Abbreviations

ATI: affinity for technology interaction
CONSORT: Consolidated Standards of Reporting Trials
COREQ: Consolidated Criteria for Reporting Qualitative Research
EFA 2.0: Elektronische FallAkte 2.0
DESAM ForNet: Initiative of German Practice-Based Research Networks
EHR: electronic health record
FA+: FallAkte Plus
GP: general practitioner
ITI: IT infrastructure
NRW-GPRN: North Rhine-Westphalian general practice research network
PBRN: practice-based research network
PrA: practice assistant
SUS: system usability scale
